# Subclavian mycotic aneurysm caused by *Aspergillus flavus* in a child with acute lymphoblastic leukemia: a case report

**DOI:** 10.3389/fped.2025.1736632

**Published:** 2026-01-26

**Authors:** Belkıs Hatice İnceli, Halil Özdemir, Elif İnce, Gül Arga, Döndü Nilay Penezoğlu, Hasan Fatih Çakmaklı, Hayreddin Aknar, Merve Havan, Meltem Koloğlu, Ömer Suat Fitöz, Evren Özçınar, Levent Yazıcıoğlu, Serpil Sak, Tanıl Kendirli, Ergin Çiftçi

**Affiliations:** 1 Department of Pediatric Infectious Diseases, Ankara University Faculty of Medicine, Ankara, Türkiye; 2 Department of Pediatric Hematology, Ankara University Faculty of Medicine, Ankara, Türkiye; 3 Department of Medical Pathology, Ankara University Faculty of Medicine, Ankara, Türkiye; 4 Department of Pediatric Intensive Care, Ankara University Faculty of Medicine, Ankara, Türkiye; 5 Department of Pediatric Surgery, Ankara University Faculty of Medicine, Ankara, Türkiye; 6 Department of Pediatric Radiology, Ankara University Faculty of Medicine, Ankara, Türkiye; 7 Department of Cardiovascular Surgery, Ankara University Faculty of Medicine, Ankara, Türkiye

**Keywords:** acute lymphoblastic leukemia, *Aspergillus flavus*, child, invasive aspergillosis, mycotic aneurysm

## Abstract

Invasive aspergillosis is a severe opportunistic infection in immunocompromised children, particularly those receiving chemotherapy for hematologic malignancies. We report the case of a 2-year-old girl with acute lymphoblastic leukemia who developed massive hemoptysis during consolidation chemotherapy. Thoracic computed tomography revealed a saccular pseudoaneurysm of the proximal left subclavian artery. Surgical resection and autologous vein graft replacement were performed, and *Aspergillus flavus* DNA was detected in the resected tissue using Aspergillus-specific polymerase chain reaction. The patient received dual antifungal therapy with liposomal amphotericin B and voriconazole, followed by long-term voriconazole prophylaxis. She made a full recovery. This case highlights the importance of considering angioinvasive aspergillosis in immunocompromised children presenting with hemoptysis and lung lesions. Early recognition and multidisciplinary management are critical to preventing fatal vascular complications.

## Introduction

Angioinvasive aspergillosis is a life-threatening opportunistic infection that predominantly affects immunocompromised children, particularly those receiving intensive chemotherapy for hematologic malignancies. Although the lungs are the primary site of infection, *Aspergillus* species possess a well-recognized capacity for angioinvasion, allowing direct extension into the pleural space, mediastinum, and, rarely, major vascular structures. Subclavian or mediastinal great-vessel involvement is exceedingly uncommon in the pediatric population, yet it carries a high risk of pseudoaneurysm formation and catastrophic hemorrhage (1).

Mycotic aneurysms account for only 0.7%–3% of all aortic aneurysms, and fungal etiologies—especially *Aspergillus*—represent an even smaller subset. Advances in cross-sectional imaging, including contrast-enhanced computed tomography (CT) and CT-angiography, have improved early detection of vascular complications in suspected invasive fungal disease. Prompt recognition is critical because pseudoaneurysms adjacent to pulmonary or mediastinal lesions may enlarge rapidly and rupture.

We describe the case of a 2-year-old girl with acute lymphoblastic leukemia (ALL) who developed a left subclavian artery pseudoaneurysm secondary to angioinvasive *Aspergillus flavus* infection during consolidation therapy. Diagnosis was supported by radiologic evidence of vascular invasion and confirmed by tissue-based *Aspergillus*-specific PCR. The patient was successfully managed using combined antifungal therapy, surgical resection with autologous vein graft reconstruction, and long-term secondary antifungal prophylaxis.

## Case report

A previously healthy 2-year-old girl presented with 1 week of progressive weakness and limping. Physical examination revealed lethargy, pallor, and widespread petechiae. Laboratory evaluation demonstrated anemia (hemoglobin 9.3 g/dL), thrombocytopenia (39,000 /mm^3^), and leukocytosis (20,000 /mm^3^) with 95% circulating lymphoblasts. Peripheral smear confirmed blasts, and bone marrow aspiration with flow cytometric analysis established the diagnosis of B-cell ALL. A port catheter was placed, and induction chemotherapy per the COG AALL-1731 protocol was initiated, with remission achieved at the end of induction.

**Figure 1:**
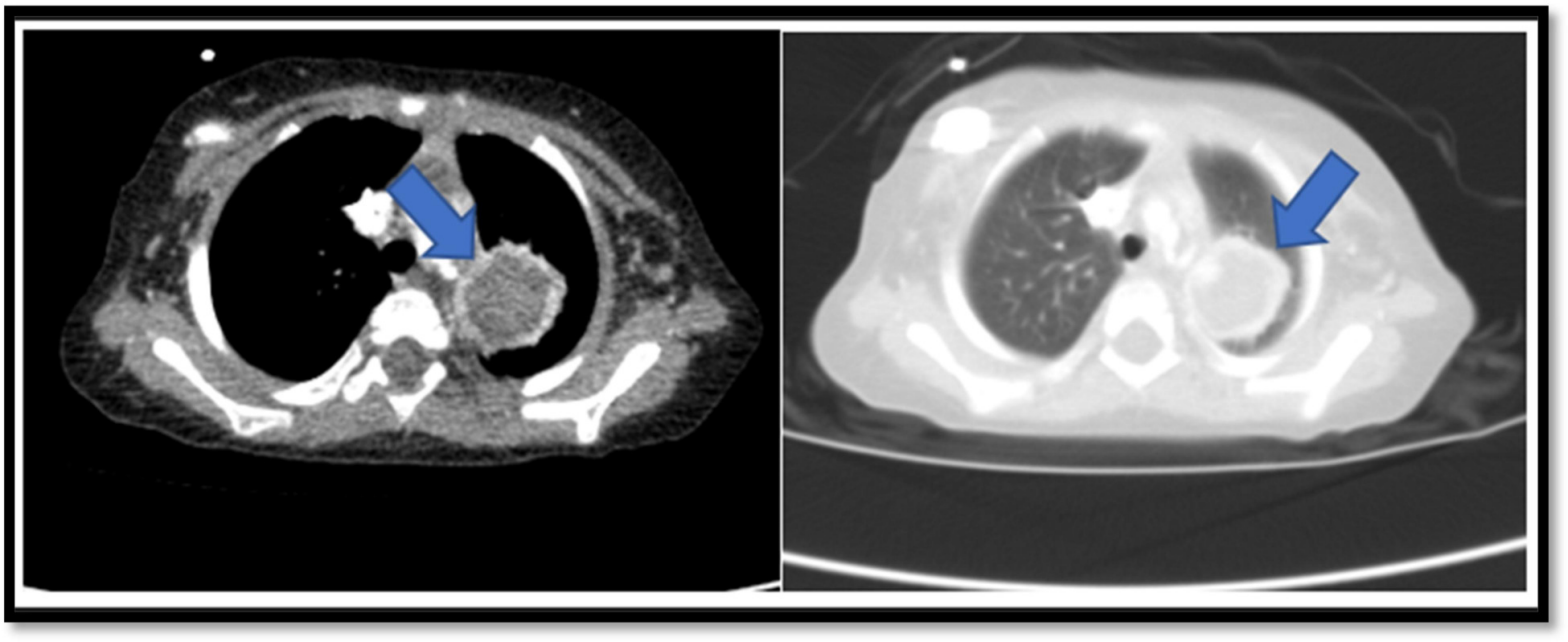
Contrast-enhanced thoracic CT showing a saccular pseudoaneurysm arising from the proximal left subclavian artery. The lesion demonstrates peripheral thrombus, extension into adjacent lung parenchyma, and associated compressive atelectasis—findings consistent with a mycotic pseudoaneurysm due to angioinvasive aspergillosis.

On day 10 of intensive consolidation therapy, the patient developed febrile neutropenia. Despite sequential broad-spectrum antimicrobials (piperacillin–tazobactam, followed by teicoplanin and levofloxacin), fever persisted. Blood, urine, stool, and throat cultures remained negative, and serum galactomannan assays were also negative. Because fever continued beyond 96 h, thoracic CT imaging was obtained, revealing a 15 × 10-mm pleura-adjacent consolidation in the left upper lobe and a 3.5-mm right lower lobe nodule with a surrounding ground-glass halo (Figure 1). These findings raised suspicion for early angioinvasive fungal infection, prompting initiation of liposomal amphotericin B (L-AmB, 3 mg/kg/day), after which the fever resolved.

On day 40 of consolidation therapy, while still receiving L-AmB, the patient developed sudden massive hemoptysis, necessitating transfer to the pediatric intensive care unit. Neutrophil and platelet counts and coagulation parameters were within normal limits, reducing suspicion of hematologic causes of bleeding. Empirical voriconazole was added, and ENT examination did not reveal an upper airway source. Contrast-enhanced thoracic CT demonstrated a saccular pseudoaneurysm arising from the proximal left subclavian artery, surrounded by thrombus, extending into the adjacent lung parenchyma, and causing compressive atelectasis. These findings were highly consistent with a mycotic pseudoaneurysm secondary to angioinvasive aspergillosis.

**Figure 2:**
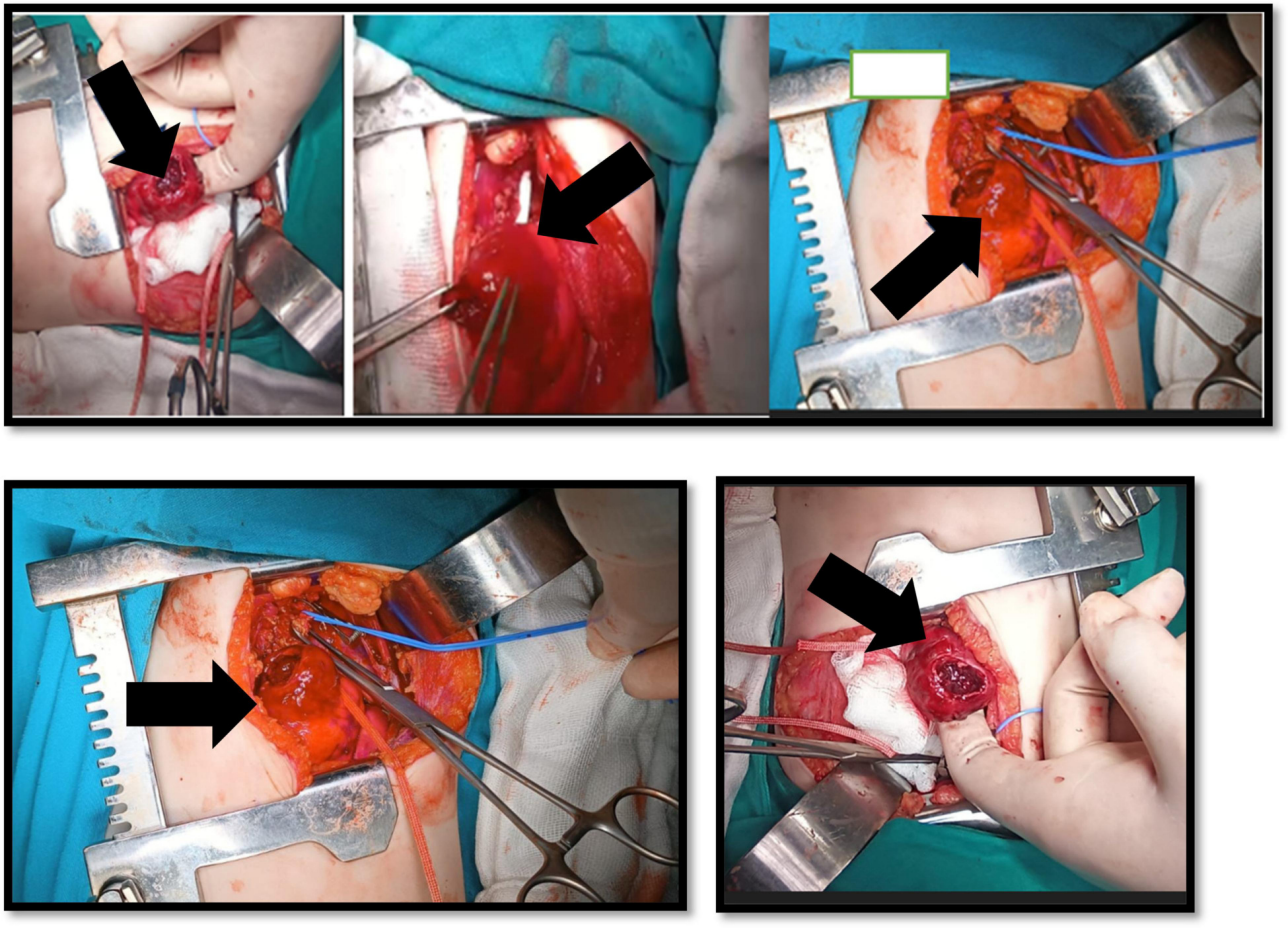
Intraoperative photograph demonstrating surgical excision of the pseudoaneurysm and reconstruction of the left subclavian artery using an autologous vein graft. Segmentectomy of the affected left upper lobe was performed due to parenchymal invasion.

A multidisciplinary meeting involving pediatric hematology, infectious diseases, pediatric ICU, pediatric surgery, and cardiovascular surgery teams concluded that urgent surgical intervention was required due to the high risk of aneurysmal rupture. The patient underwent left upper lobe segmentectomy with complete excision of the pseudoaneurysm, and the left subclavian artery was reconstructed using an autologous vein graft (Figure 2). Gross pathology revealed consolidated lung parenchyma with an 18-mm cavitary lesion.

**Figure 3:**
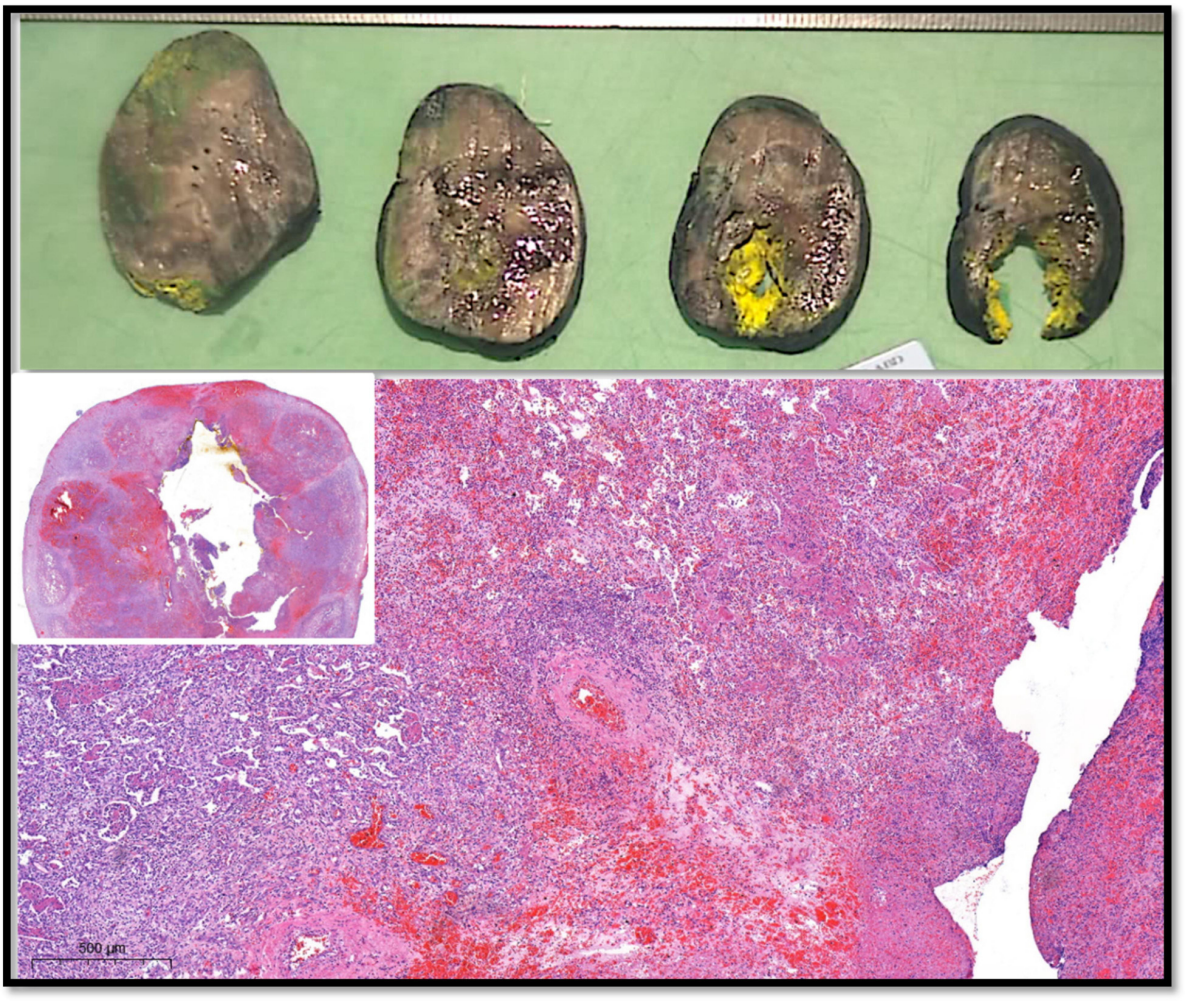
Histopathological sections of the excised lung tissue. Hematoxylin–eosin staining shows hemorrhage, necrosis, and intense suppurative inflammation surrounding the cavitary lesion. PAS and Grocott methenamine silver stains did not demonstrate fungal hyphae, consistent with treated angioinvasive aspergillosis and tissue necrosis.

Microbiologic evaluation showed no growth in tissue cultures, and serum galactomannan remained negative. Histopathologic examination demonstrated hemorrhage, necrosis, and suppurative inflammation, but no fungal elements were visible on periodic acid-schiff (PAS) or Grocott methenamine silver (GMS) staining—a well-recognized limitation in cases of angioinvasive aspergillosis following antifungal exposure or significant tissue necrosis (Figure 3). However, *Aspergillus*-specific PCR performed on excised tissue was positive for *A. flavus*, confirming the etiologic organism. Based on host factors (ALL with neutropenia), radiologic evidence of vascular invasion, and positive fungal PCR, the case fulfilled European organization for research and treatment of cancer (EORTC)/mycoses study group (MSG) criteria for probable invasive aspergillosis (IA) with vascular involvement.

Postoperative CT angiography showed complete resolution of the pseudoaneurysm. Low-dose aspirin (5 mg/kg/day) was initiated to prevent graft thrombosis and was recommended as lifelong therapy by the cardiology and cardiovascular surgery teams. Dual antifungal therapy with L-AmB and voriconazole was continued for 60 days, and long-term voriconazole prophylaxis was maintained for 17 months throughout consolidation and maintenance therapy. Discontinuation was considered once imaging remained stable and the patient reached day 71 of the third maintenance cycle.

## Discussion

IA remains one of the most severe infectious complications in immunocompromised children, particularly those receiving intensive chemotherapy for hematologic malignancies. Although classic risk factors include prolonged neutropenia, high-dose corticosteroid exposure, hematopoietic stem cell transplantation, and intensive immunosuppressive therapy, recent evidence indicates that additional factors such as viral infections (including influenza and SARS-CoV-2), prolonged ICU stays, and the wider use of newer chemotherapeutic agents further heighten susceptibility to IA. Despite advances in medical therapy, pediatric IA continues to carry a substantial mortality risk, underscoring the importance of early identification and aggressive management (2).

In most patients, IA begins with inhalation of *Aspergillus conidia*, leading to pulmonary or sinonasal infection. Angioinvasion is a hallmark of severe disease and can result in extrapulmonary dissemination. While vascular invasion is well described in adults—particularly involving the aorta and major thoracic vessels—reports of mediastinal or subclavian artery involvement in children are exceedingly rare. The ability of *Aspergillus* to invade vessel walls can lead to pseudoaneurysm formation, thrombosis, and catastrophic hemorrhage. In our patient, massive hemoptysis served as the sentinel event uncovering a subclavian artery pseudoaneurysm—a previously unreported presentation in a child with ALL undergoing consolidation chemotherapy (3).

Children with ALL are particularly vulnerable to IA during phases of intensive treatment, especially consolidation, when neutropenia and corticosteroid exposure converge. This is consistent with the clinical course of our patient, who developed IA during consolidation. Although antifungal prophylaxis remains controversial in newly diagnosed ALL, emerging ECIL-10 guidelines recommend prophylaxis for selected high-risk patients, particularly those with poor early response, persistent neutropenia, or intensified chemotherapy protocols. Our patient was not receiving primary prophylaxis at the time the infection developed, which is consistent with common practice in standard-risk ALL (4, 5).

Once IA was suspected, therapy was initiated with L-AmB and voriconazole. The combination aligns with infectious diseases society of America (IDSA) and European conference on infections in leukemia (ECIL) guidance, which recognize voriconazole as first-line therapy and L-AmB as an acceptable alternative or adjunct in severe cases. Voriconazole therapeutic drug monitoring was unavailable; however, the patient was monitored clinically and biochemically, and no evidence of toxicity or treatment failure emerged. Importantly, voriconazole's CYP3A4 inhibition raises concerns in pediatric ALL because of potentiation of vincristine neurotoxicity. In this case, close monitoring allowed continuation of chemotherapy without enhanced toxicity, and the addition of L-AmB mitigated pharmacokinetic limitations (5–7).

The detection of a subclavian pseudoaneurysm raised critical management considerations. Although endovascular repair is increasingly common in adults, open surgical repair remains the preferred approach in children due to anatomical constraints, graft durability concerns, and the need for thorough debridement of infected tissue. In our case, multidisciplinary consensus favored open resection with graft replacement, which provided definitive control of infection and prevented potential arterial rupture (8).

Histopathologic examination did not demonstrate fungal elements on PAS or GMS staining, a finding not uncommon in angioinvasive aspergillosis. Prior antifungal therapy, tissue necrosis, and sampling variability may all contribute to false-negative histopathology. Molecular analysis using *Aspergillus*-specific PCR, however, confirmed *Aspergillus flavus* DNA within the resected tissue. Although galactomannan was negative, this is compatible with vascular-restricted aspergillosis, where circulating antigen levels may be low. Taken together, the combination of host factors, radiologic evidence, vascular pathology, and positive tissue PCR satisfied the EORTC/MSG criteria for probable IA.

This case emphasizes that sudden hemoptysis in an immunocompromised child should prompt evaluation for angioinvasive fungal disease with possible vascular involvement. Early imaging, interdisciplinary collaboration, and timely surgical intervention are essential to prevent fatal hemorrhage. Further, updated guideline recommendations underscore the need for individualized antifungal prophylaxis strategies and highlight the importance of understanding drug–drug interactions when selecting antifungal agents in pediatric ALL.

In conclusion, this case illustrates the need for early recognition of rare but life-threatening vascular complications of invasive aspergillosis in immunocompromised children. Persistent fever, pulmonary nodules, and especially hemoptysis in ALL patients should raise suspicion for angioinvasive aspergillosis with vascular involvement. Successful management requires multidisciplinary collaboration. Furthermore, updated ECIL-10 guidelines stress the importance of tailoring prophylaxis strategies in high-risk ALL patients, while careful attention to antifungal Drug-Drug Interactions (DDIs) remains essential.

## Data Availability

The raw data supporting the conclusions of this article will be made available by the authors, without undue reservation.
